# Gut microbiota shifts in spaceflight: a case study evidence and countermeasures for microbial homeostasis

**DOI:** 10.1007/s13205-026-04760-9

**Published:** 2026-03-31

**Authors:** Raagul Seenivasan, Jey Kumar Pachiyappan, Anitha Marimuthu, Praveen Halagali, Gowthamarajan Kuppusamy, Pawan Ganesh Nayak, Vamshi Krishna Tippavajhala

**Affiliations:** 1 Department of Pharmaceutics, Manipal College of Pharmaceutical Sciences, Manipal Academy of Higher Education, Manipal, India; 2 Department of Pharmaceutics, JSS College of Pharmacy, JSS Academy of Higher Education and Research, Ooty, The Nilgiris, 643001 Tamil Nadu India; 3 Department of Pharmacology, JSS College of Pharmacy, JSS Academy of Higher Education and Research, Ooty, The Nilgiris, 643001 Tamil Nadu India; 4 Department of Pharmacology, Manipal College of Pharmaceutical Sciences, Manipal Academy of Higher Education, Manipal, India

**Keywords:** astronauts, gut microbiota, microgravity, microbial dysbiosis, Microbial homeostasis, space microbiome

## Abstract

The gut microbiota is a crucial component in maintaining overall human health since it has been found to influence not only metabolism but also neurobehavioral function and immunity. The extreme conditions of space, for example, cosmic radiation, microgravity, and confinement, can severely disrupt the functioning and alter the composition of gut microbiota. In fact, this will predispose the immune system to be dysfunctional, lead to psychological and metabolic disorders that are accompanied by a decrease in the diversity of beneficial microbes and change in the pattern of metabolite production. The spaceflight analog and ground, based studies have produced important findings concerning the mechanisms and reasons for gut microbial dysbiosis in extreme conditions. Different research works have been carried out, such as dietary intervention and high fiber to support the growth of healthy microbes. Further, advanced microbial monitoring using wearable sensors to identify the microbial and proinflammatory biomarkers will mitigate dysbiosis and safeguard the crew’s health for longer-duration missions. This wearable sensor will not only help monitor astronauts’ microbial status continuously, but it will also provide a significant feature for designing personalized dietary plans and probiotic supplements. This article provides a comprehensive understanding of astronaut health, including disturbances to the gut microbiome during space travel, space-analogue studies conducted by many researchers to unravel mechanisms, countermeasures to stabilize the gut microbiome, and its prospects.

## Introduction

Space travel began in the 1960s, and in recent years, space exploration has become more popular. Astronauts undergo a variety of physiological and biological changes that impact their health. These changes are mainly linked to space-specific environmental factors, such as microgravity, disrupted circadian cycles, exposure to cosmic radiation, decreased physical activity, and confined-space factors, which become particularly significant during extended space missions (Landry et al. 2020; Krittanawong et al. 2022; Gimunová et al. 2024). Astronauts on deeper space missions have been identified with skeletal muscle atrophy (Liu et al. 2024), osteoporosis (due to excessive excretion of calcium and phosphorus) (Banimohamad-Shotorbani et al. 2024), body fluid redistribution (Noskov 2013), cardiopulmonary deconditioning (Ahmed et al. 2024), Immune dysfunction (Sun et al. 2021), and alteration in the microbial fauna (Ibrahim et al. 2024). Various research studies have been conducted in simulated microgravity conditions on Earth better to understand the impact of microgravity on gut microbiota. Using the biorthogonal metabolic labelling method, rats were exposed to microgravity and monitored non-invasively (Thiel et al. 2021). Rats experienced tissue damage and elevated inflammatory markers due to increased gut microbiota, leading to increased plasma endotoxin levels and intestinal barrier dysfunction. Another study examined pathophysiological changes in a mouse model by exposing it to high linear energy transfer (LET) radiation. It has been observed that there is an increase in opportunistic bacteria and a decrease in gut microbiota; further, LET radiations may induce functional shifts in metabolism (Suzuki et al. 2024). Yifan et al. studied the relationship between altered glucose metabolism and gut dysbiosis using the hind-limb unloading mouse model. Plasma LPS-binding protein levels increased, and transcription of TNF-α and glucose metabolism-related genes was altered. Additionally, the HU mouse model exhibited Impaired Body weight gain, peripheral insulin resistance, reduced gut microbiota (Bifidoba*cterium* spp. and *Akkermansia muciniphila*), and glucose intolerance (Wang et al. 2019).

Studies conducted during space missions assessed the gut microbiome of mice using metagenomics before and after spaceflight. Bacteria linked to fatty acid and bile metabolism were identified in microgravity, and there were significant changes in gene expression related to microbial interactions, barrier functions, and bile acid transport (Wang et al. 2024). Braden T. Tierney et al. conducted a longitudinal study to quantify human microbiomes over 3 days in 4 individuals. 750 samples were collected during and after space flights from 10 body sites, and they were characterised using paired metagenomics and meta transcriptomics alongside single-nuclei immune cell profiling, revealing that astronauts experienced immune response shifts during short-term space missions (Tierney et al. 2024). Astronauts going on missions further into space are exposed to quite a number of physiological and pathological changes as a result of radiation and microgravity conditions, which have a substantial impact on their health. According to the research carried out in the last decade, microgravity changes the composition of the gut microbiota causing microbial dysbiosis (imbalance between beneficial and harmful bacteria), the rise of opportunistic bacteria, and the weakening of immune and autoimmune responses. The gut barrier integrity is compromised, thereby increasing intestinal permeability (leaky gut) and astronauts becoming more susceptible to infections and inflammation (Mortazavi et al., 2024).

Furthermore, microgravity conditions alter the production of short-chain fatty acids (SCFA) by microbes, impairing the digestion and absorption of nutrients during deeper space missions (Turroni et al. 2020). Continuous exposure to cosmic radiation damages the DNA of human cells and gut microbes, creating oxidative stress (Zhang et al. 2025). Prolonged stay in the confinement environment causes psychological stress to the astronauts, which in turn alters the gut microbiota and disrupts the gut-brain axis, leading to mood disorders, cognitive impairments, and neuroinflammation. For the proper functioning of drug-metabolising enzymes, gut microbiota plays a crucial role. Alterations in the diversity and health of the gut microbiota directly or indirectly affect the drugs that are metabolised by these enzymes—E*ggerthella lenta* spp., which is present in the intestines, the gut microbiota has been shown to modify Digoxin (used for Atrial Flutter, Atrial fibrillation, and heart failure); during space missions, modifications in the gut microbiota have the potential to cause sub-therapeutic levels or digoxin toxicity (Pachiyappan et al.,^2024^).

Significant changes are brought in the gut microbiota’s composition and functions, leading to microbial dysbiosis, leaky gut, and immune and metabolic dysfunction because of microgravity, radiation, and psychological stress, impacting the astronaut’s health negatively by making them susceptible to infections, inflammations, and cognitive impairments (Nie et al. 2024). Understanding these mechanisms will help mitigate these issues and ensure astronauts’ health during deeper space missions. Exploration of human health during space travel has increased, but the specific impact of microgravity on gut microbiota remains unexplored. Earlier studies have shown variations in the gut microbiota of astronauts; however, they are lacking in providing a comprehensive picture of the ways that these changes happen and what their effects might be on the astronauts in the long run. At present, research is mainly focused on microbial changes, immune modulatory effects, and metabolic variations separately, thus researchers are not yet integrating these three components into one comprehensive model (Siddiqui et al. 2024). This paper aims to provide details on how space gravity impacts the composition and function of the gut microbiota, analysing spaceflight and ground-based analogue studies and their consequences for astronaut health during deeper space missions.

Additionally, the review gives a comprehensive knowledge of countermeasures in maintaining gut microbiota balance in microgravity and its significance. Various strategies, such as the intake of pre- and probiotics, psychological stress management, radiation shielding, and simulated gravity conditions, have been discussed to counterbalance the negative impact of space travel. It also covers the inevitable need for future research on personalised microbiome-based diets, bio-engineered gut microbes, and real-time monitoring of microbes in space. Overall, this paper paves the way for further exploration of the gaps and provides innovative methodological paradigms, while emphasising the need to integrate microbiology and space medicine.

## Health implications of gut mcrobiota sysbiosis

### Microbial dysbiosis

**Fig. 1:**
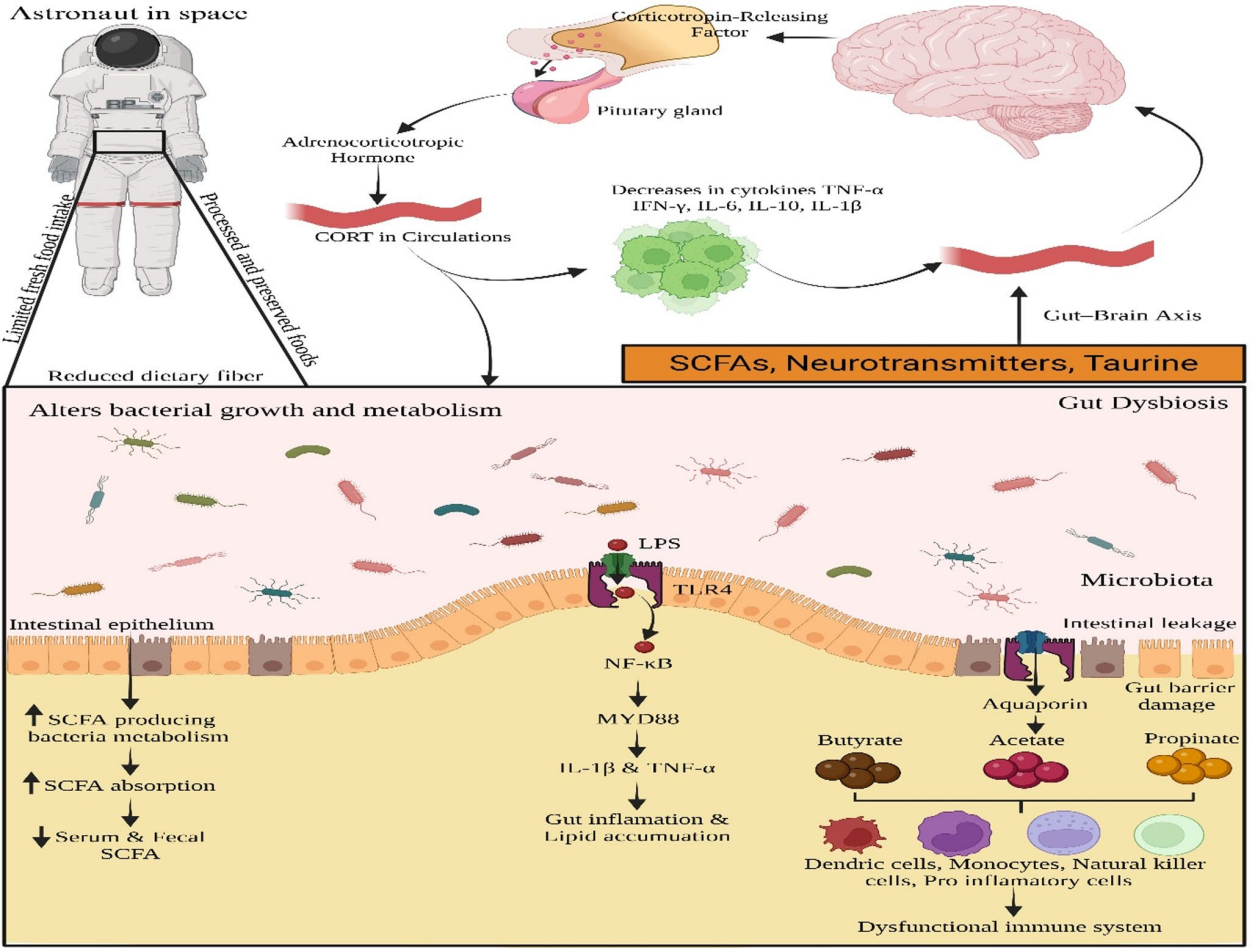
Microbial Dysbiosis Induced by Microgravity (Created using https://www.biorender.com)

Radiation & Microgravity Exposure will disrupt gut microbiota homeostasis by increasing commensal bacteria, such as Lactobacillus and Bifidobacterium, and reducing pathogenic bacteria, such as Escherichia, Clostridium, and *Enterococcus* (Jian et al. 2021; Zhao et al. 2021; Koehle et al. 2023). This led to the disruption of Short-Chain Fatty Acids (SCFAs), such as acetate, propionate, and butyrate, produced by gut bacteria during the fermentation of dietary fibres and which contribute to various physiological functions. Acetate is more abundant and is produced by *Bifidobacteria*,* Akkermansia*, and some *Firmicutes* species. It serves as a precursor for lipogenesis (fat synthesis) and regulates host metabolism through G-protein-coupled receptors (GPR43 & GPR41) (Ríos-Covián et al. 2016; Koh et al. 2016). Bacteroidetes, Veillonella, and some other Firmicutes species produce propionate. It is metabolised by the liver for gluconeogenesis and inhibits cholesterol synthesis. It also regulates appetite and energy balance by stimulating GLP-1 (Glucagon-like peptide-1) release. It acts as an anti-inflammatory agent by suppressing pro-inflammatory cytokines. Butyrate is produced by *Firmicutes* such *as Faecalibacterium prausnitzii*,* Roseburia*,* and Clostridium* spp. It reduces the inflammation by inhibiting NF-κB signalling and enhances intestinal epithelial cell differentiation and repair (Parada Venegas et al. 2019). The TLR4 & NF-κB Pathways were activated, leading to the production of pro-inflammatory cytokines (IL-6, TNF-α) (Reichardt et al. 2014; Amiri et al. 2022; Singh et al. 2023). This leads to increased gut inflammation, alters nutrient metabolism, affects glucose homeostasis, and results in an increase in oxidative stress (Clemente et al. 2012; Shin et al. 2015). As shown in Fig. 1, microgravity induces microbial dysbiosis via multiple mechanisms, resulting in significant alterations in host physiology.

### Increased gut permeability (leaky gut)

Increased gut permeability, or leaky gut, is observed in astronauts due to factors such as microbiome dysbiosis, disruption of tight junction proteins, oxidative stress and inflammation, immune dysregulation, and systemic effects, as well as mucosal layer degradation (Alvarez et al. 2019; Turroni et al. 2020). Microbiome dysbiosis will occur due to alterations in the composition and diversity of the gut microbiota, and to decreases in butyrate-producing bacteria such as Faecalibacterium prausnitzii and Roseburia, which weaken the intestinal epithelial barrier. This alteration in the microbiome also leads to inflammation and endotoxin (LPS) production, thereby damaging the gut lining (Derrien and van Hylckama Vlieg 2015; Oliphant and Allen-Vercoe 2019; Kurilshikov et al. 2021). Disintegration of tight junction proteins can diminish the gut barrier’s efficacy and raise its permeability. The mechanism involved here is the activation of NF, B, which results in inflammation and the subsequent degradation of tight junction proteins. Hence, the compromised gut barrier permits the entry of bacteria and toxins into the bloodstream, causing systemic immune reactions (Zhang et al. 2021; Li et al. 2024, 2025b; Neurath et al. 2025). Galactic environments elevate ROS synthesis in intestinal cells, which in turn harms epithelial cells and tight junction proteins. It likewise causes the release of pro, inflammatory cytokines like TNF, and IL, 6, that in turn break down tight junction proteins and make the gut more permeable (Al-Sadi et al. 2013, 2016; Capaldo et al. 2014; Xiao et al. 2016). Oxidative damage weakens gut integrity, making astronauts more susceptible to infections and chronic inflammation (Yang et al. 2020; Tesei et al. 2022; Capri et al. 2023; Dakkumadugula et al. 2023; Marchal et al. 2024). The gut mucus layer consists of mucin proteins. These mucin proteins are affected by microgravity exposure, thus leading to bacterial invasion and inflammation (Christovich and Luo 2022; Poto et al. 2023).

### Immune system suppression

The gut microbiome produces several molecules, such as TLR/NOD ligands, SCF, and AhR ligands, which interact with gut lining cells (enterocytes) and immune cells. These molecules enter the systemic circulation and affect the immunity in other organs. There is evidence that exposure to microgravity alters the gut microbiota’s composition and function, leading to impaired immunological signalling and dysregulation of microbial metabolites(Turroni et al. 2020; Wang et al. 2020a; Tesei et al. 2022; Ibrahim et al. 2024). Foxp3 + Treg, Tfh/exTh17 are the immune cells present in Peyer’s patches that help B cells switch to producing secretory IgA (sIgA) that maintains the microbiota balance and prevents the multiplication of harmful bacteria. Th17 cells are another type of immune cell that are made by Segmented Filamentous Bacteria (SFB) and other commensal bacteria. Moreover, SFB induces ILC3 cells to release IL-22, which in turn induces Th17 cells to produce IL-17 A. This keeps certain bacteria under control and inhibits them from proliferating (Hirota et al. 2013; Zheng et al. 2020; Heidari et al. 2024). There is a special type of protein called MHC II, which is mainly found in ILC3 cells and helps prevent CD4 + cells from affecting harmless gut bacteria. This intestinal bacterium plays a role in balancing Th1 and Th2 cells and influencing CD4 + T cell differentiation. Immune cells (DCs) via TLR2 internalize a molecule from B. fragilis called PSA (polysaccharide A). iTreg cells may be induced to arise in the presence of TGF, and generate IL, 10 for immune homeostasis, while IL, 23 favors the proliferation of Th17 cells causing inflammation. The microbiota communicates the immune system and collaborates with it. It does so mainly by regulating the immune cell balance and guard against unnecessary assaults on non, pathogenic microbes. Alterations of the gut microbiota due to microgravity have the potential to compromise the immune system’s normal regulatory functions. Thus, the issue of sustaining a reliable microbial equilibrium in space missions becomes even more critical. iTreg cells may be induced to arise in the presence of TGF, and generate IL, 10 for immune homeostasis, while IL, 23 favors the proliferation of Th17 cells causing inflammation. The microbiota communicates the immune system and collaborates with it. It does so mainly by regulating the immune cell balance and guard against unnecessary assaults on non, pathogenic microbes (Yoo et al. 2020; Liu et al. 2022; Magalhães et al. 2025). Alterations of the gut microbiota due to microgravity have the potential to compromise the immune system’s normal regulatory functions. Thus, the issue of sustaining a reliable microbial equilibrium in space missions becomes even more critical (Crucian et al. 2008; Cervantes and Hong 2015; Al et al. 2022; Wadhwa et al. 2024; Etlin et al. 2024).

### Disrupted gut-brain axis

Bacteria in the gut ferment fibres and produce short, chain fatty acids (SCFAs) such as acetate, propionate, and butyrate. These SCFAs are the main energy source for colon cells and the immune system. In addition to this role, SCFAs contribute to maintaining the intestinal barrier integrity, regulate gut hormones, and prevent oxidative damage of cells (O’Riordan et al. 2022; Ma et al. 2022; Hays et al., 2024). A study showed that microgravity reduces the abundance of short, chain fatty acid (SCFA) producing bacteria, which in turn weakens the gut barrier function and alters the production of key microbial metabolites (Wu et al. 2024a; Xiong et al. 2025; Sun et al. 2025). SCFAs also control gene expression and long, term biological functions through histone modification. Besides, SCFAs can also cross the blood, brain barrier and have an impact on brain function. Butyrate is an anti, inflammatory agent that functions by activating regulatory T cells to release interleukin, 10. These changes maintain a balanced immune response and also protect the brain, axis integration (Nshanian et al. 2025; Li et al. 2025a; Kabir et al. 2025; Wachamo and Gaultier 2025). Studies show that microgravity, induced gut disorders can change the concentration of neuronally active short, chain fatty acids (SCFAs), thus possibly leading to disrupted mood regulation and enhanced neuroinflammation (Cheng et al. 2024; Wang et al. 2024; Wu et al. 2025; Yang et al. 2025a). Therefore, a healthy gut microbiome can be achieved by eating fibre, rich foods and taking probiotics, which in turn may help to keep balanced levels of SCFA and support mental health (Cheng et al. 2024; Basnet et al. 2024; Shaikh et al. 2025). Bile salts are synthesized in the liver and the intestine; the gut bacteria then transform primary bile acids into secondary bile acids. This conversion of bile acids is crucial for the maintenance of a healthy gut environment (Winston and Theriot 2020; Afzaal et al. 2022; Wise and Cummings 2023). One of the ways bile salts affect the central nervous system (CNS) is by binding to neurotransmitter receptors such as the M2 and M3 muscarinic acetylcholine receptors, GABA receptors, and NMDA receptors. According to the work on hypothalamic neurons, primary bile acid chenodeoxycholic acid (CDCA) inhibits GABA and NMDA receptor activities that regulate brain functions (Schubring et al. 2012; Romanazzi et al. 2023). Spacefaring conditions including microgravity, can alter the biochemical process of secondary bile acids formation resulting in a disruption of neurotransmitter receptor signal transduction and brain function. Hypo, production of secondary bile acids might cause gut microbiota dysbiosis, elevated intestinal permeability, and inflammation that may lead to anxiety. Besides taking a nutritionally rich diet replenishing gut probiotic flora will significantly improve psychological wellbeing. (Clapp et al. 2017; Yuan et al. 2023; Lawn et al. 2023; Kyei-Baffour et al. 2025). High concentrations of pro, inflammatory cytokines, such as Interleukin, 1 beta, IL, 2, IL, 6, IL, 8, IL, 12, IFN, and TNF, may result in various neurodegenerative disorders (Zhang et al. 2023; Tylutka et al. 2024; Mallick et al. 2025). Lipopolysaccharides (LPS) are bacterial endotoxins that cause pro, inflammatory reactions and thus reduce thymus weight. Moreover, LPS leads to an increase in Interferon gamma, Interleukin 10, superoxide, and corticosterone levels (Gruver and Sempowski 2008; Ullewar and Umathe 2014; Luo et al. 2021; Collins et al. 2024). LPS can translocate from the intestinal lumen to the systemic circulation through the intestinal barrier and may induce neurological impairment (Lu et al. 2022; He et al. 2024). It has been reported that exposure to microgravity results in a compromised gut barrier that allows lipopolysaccharides (LPS) to leak into the blood, which may lead to brain inflammation and cognitive and behavioural impairment.

## Spaceflight analogue studies

### Hindlimb Unloading Model

**Fig. 2:**
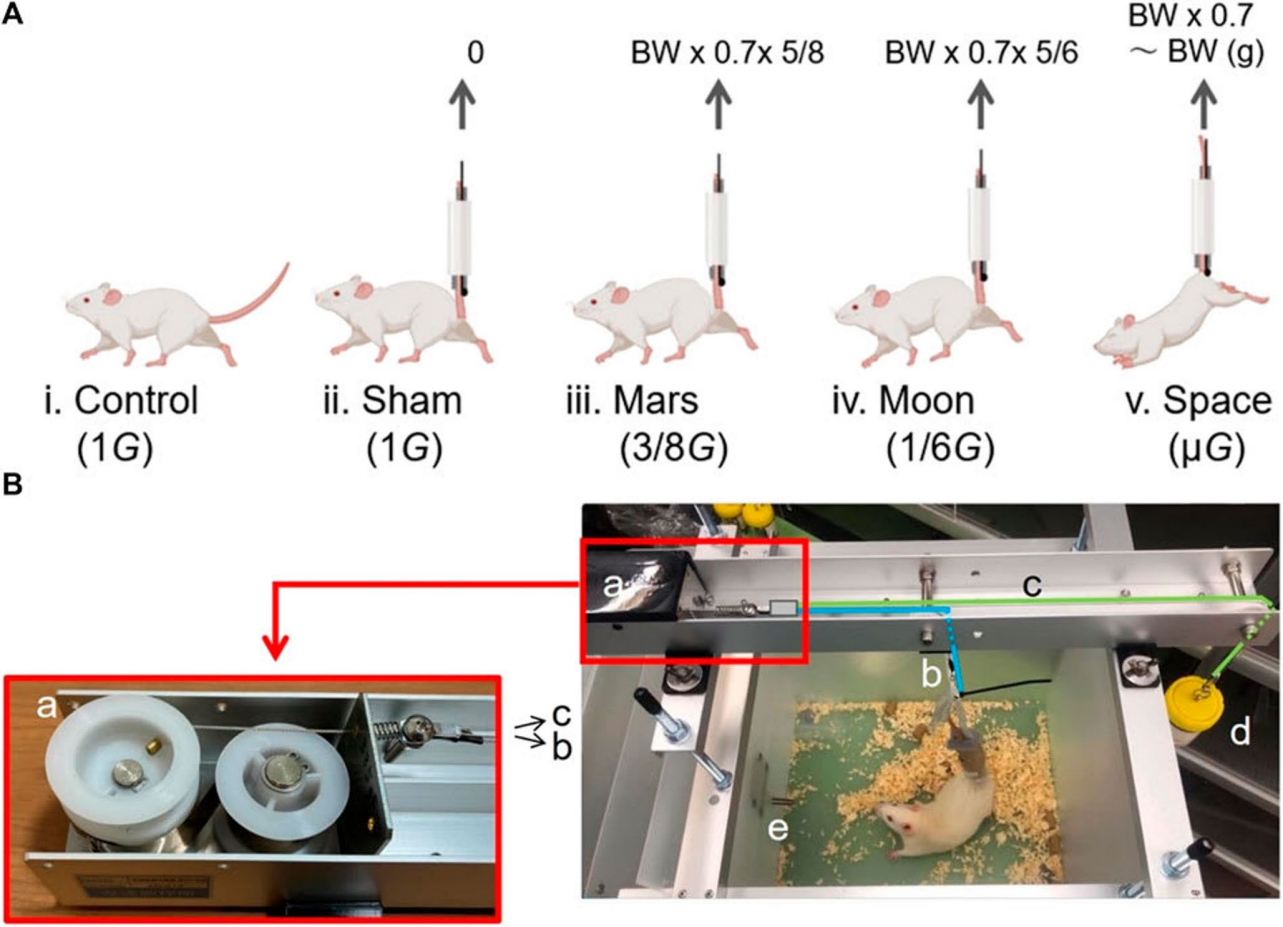
(A) The groups of experimental rats (B) simulated partial gravity apparatus. a. Black Spring storage box with two built-in pulleys and a spring wire splitter; B.b. spring wire connected to the rat’s tail; B.c. spring wire connected to the balance container; B.d. balance container; and B.e. partition (Globus and Morey-Holton 2016; Nday et al. 2019; Zhang et al. 2022)

The apparatus consisted of a long stainless-steel slot with four brackets attached to a specialised cage. The key part is a fixed slot holding a system with five components: a black spring storage box with pulleys, two spring wires (one connected to the rat’s tail and the other to a balance container), a balance container to control force, and a partition to separate the water bottle from the rat’s space. The spring wires are joined via a splitter which permits movement. One wire elevates the rat’s tail, thus the hindlimbs are kept off the cage floor, while the balance container acts against this force, hence adjusting the levels of gravity. Once the container is put on, it moves the splitter so that the rat is lowered and therefore can use all four limbs to eat and drink. The weight placed in the balance container is figured out from the rat’s weight and thus proper simulation of Moon or Mars gravity is ensured by the adjustment of lead weights. The detailed overview of the instrument is depicted in Fig. 2 (Globus and Morey-Holton 2016; Nday et al. 2019; Zhang et al. 2022).

### Bed Rest Studies

**Fig. 3:**
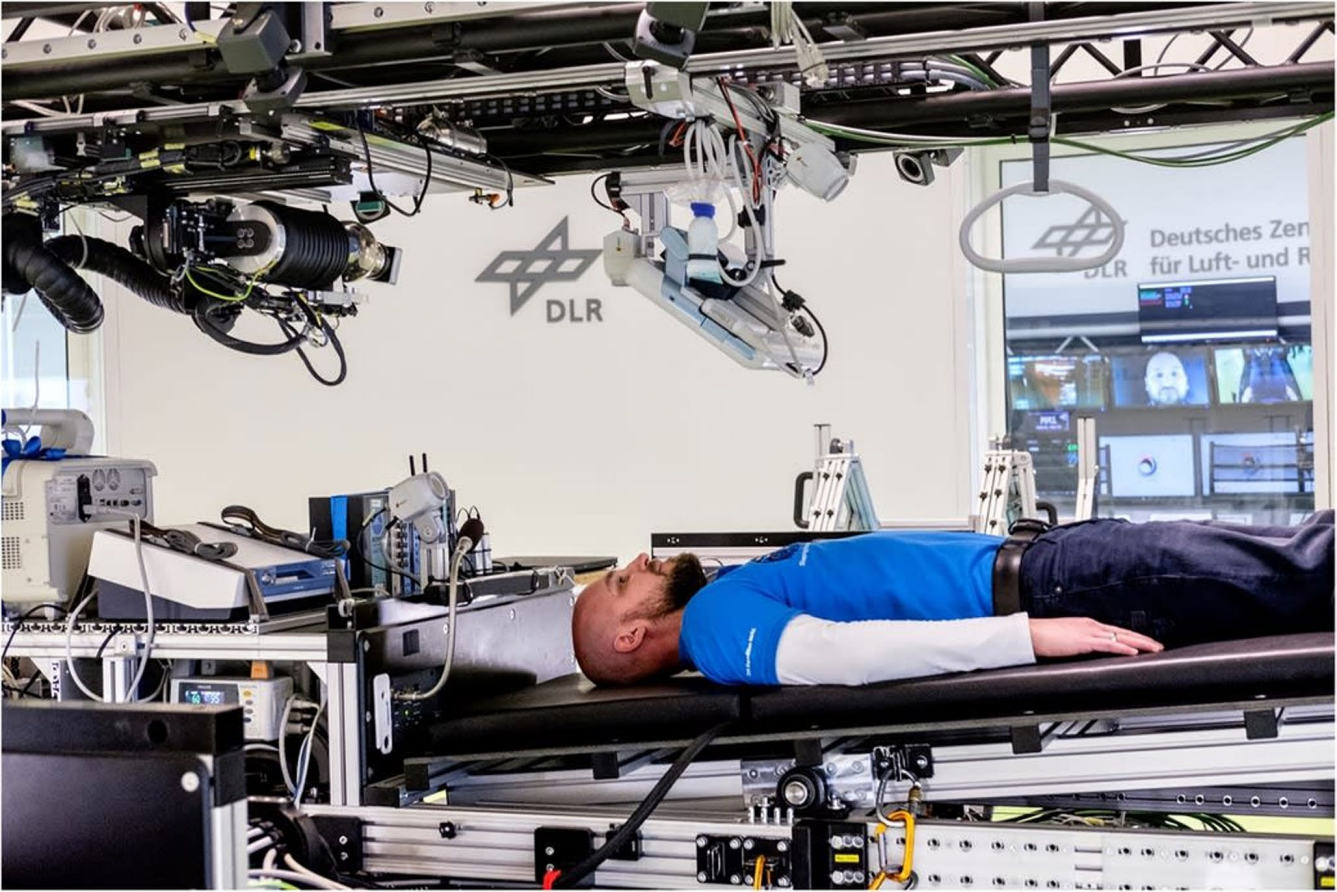
Envihab research facility. Participants were placed in the centrifuge with their heads facing the center. One Gz was to be achieved at the centre of mass (Hoenemann et al. 2023)

Bed rest studies examine the effects of microgravity on humans in a simulated environment. Initially, horizontal bed rest was followed, but Russian scientists later introduced a 6° head-down tilt (HDT) position that can accurately mimic the headward fluid shifts observed in space, in which blood moves from the lower body to the upper body. Recently, the HDT bed rest model has become the most common method for studying microgravity-related physiological changes. Long-term bed rest studies, lasting from minutes to a year, are conducted with healthy volunteers to investigate the effects of microgravity on bones, muscles, and the cardiovascular system, and to evaluate potential countermeasures. Participants are continuously monitored and maintained in controlled conditions, including a regulated diet, physical activity, fluid intake, stress levels, and light-dark cycles. This is a typical model for studying aspects of the body affected by spaceflight such as cardiovascular deconditioning, lowered exercise capacity, and the breakdown of the musculoskeletal system. It is likewise a tool for trying out the ways of preserving muscle and bone, as well as the heart, and preventing orthostatic intolerance. Since the times when human Mars exploration was hinted, there have been reports claiming that emotion and sleep behavior in astronauts are the most vulnerable to isolation and confinement in space. To validate such claims, bed rest studies were utilized to simulate those factors (Kehler et al. 2019; Ferranti et al. 2020). Figure 3 depicts the bed rest study conducted at the Envihab research facility, which simulated microgravity conditions (Hoenemann et al., 2023).

## Ground-based simulation studies on gut microbiota

The following case studies detail the impact of simulated microgravity conditions on the gut microbiota and how these alterations or imbalances affect human health, especially the space travellers. Several researchers have utilised different microgravity simulation approaches to mimic the space environment, including the Hind limb tilt method, rotating wall vessel, simulated space capsule, and bed rest method. Various gut microbial species affected by these simulated conditions were also discussed in the following sections. Various researchers also studied the effects of radiation on gut microbes and have reported an evidence-based conclusion.

### Microgravity Effects

Sarah Castro-Wallace et al.(Castro-Wallace et al. 2017) studied probiotic *Lactobacillus acidophilus* (LA) under simulated microgravity conditions using a rotating wall vessel and compared the results with normal ground state conditions. The results from these environments were compared for growth, gene activity, and stress resistance. Several researchers have hypothesised that consuming probiotics during space travel may improve gut health, but it is necessary to assess their effectiveness before their use. LA was selected for this study because it is widely used and offers various benefits, including destroying harmful bacteria, boosting immunity, preventing diarrhoea, and reducing infections. In this study, the probiotics are grown under microgravity without oxygen. This LA was cultivated under simulated low shear-modelled microgravity conditions. The bioreactors are attached to the rotating wall vessel, which rotates to minimise turbulence and average the gravitational vector, thus creating a “Low shear condition” that mimics the space environment. In this study, the researchers monitored the growth of the microbes over 16 h by collecting samples at different time points, and pH was measured at the end of the experiment. These bacteria were also exposed to simulated gastric and intestinal pH conditions. The samples were collected, washed, and tested to survive for 2 h. The number of living cells was compared with that of untreated controls. RNA sequencing was performed to analyse gene expression by extracting and purifying RNA, removing ribosomal RNA, preparing sequencing libraries, and analysing differential gene expression using bioinformatics tools. The results revealed that LA cultured under microgravity and control conditions showed similar growth patterns. The final pH was also comparable, and no significant differences in survival were observed when stationary-phase cultures were exposed to simulated gastric or intestinal juices. RNA-Seq revealed no significant differences in gene expression between microgravity and control cultures, and transcriptomic profiles were also highly correlated. Finally, the results showed that there was no significant change in LA when it was exposed to both environmental conditions.

Joseph K. Bedree et al. (2023) studied the link between gut microbes and bone loss under microgravity conditions. The gut microbiota was studied during the NASA Rodent Research 5 mission. The researchers have found that certain bacteria, such as L. murinus and Dorea sp., were more abundant in space. These produced higher concentrations of metabolites, including lactic acid, amino acids, and glutathione, in the blood of space-exposed mice. There was also bone density loss due to elevated osteocalcin levels and lower levels of enzymes such as tartrate-resistant acid phosphatase. Research in this area is limited due to the short duration of missions and the small sample size available for study. To overcome these limitations, NASA developed a rodent habitat that enables multiple samplings over a more extended period. The research investigates the relationship between bone health and microbiomes (oral and gut). According to this research, immunological modulation, food absorption, and metabolic activity are among the ways in which gut microorganisms influence bone regulation. It is known that gut bacteria-produced short-chain fatty acids influence bone metabolism by lowering markers of bone breakdown and increasing insulin-like growth factor-1.

DNA was extracted from the faecal and oral samples, and these were sequenced using Illumina platforms. The data were pre-processed to remove noise in the sample. The taxonomy was assigned using the SILVA, HOMD, GreenGenes Gold, and NCBI databases. The alpha and beta diversity analyses were performed using statistical tools to identify significant bacterial variation and to develop heat maps. Whole-genome sequencing was performed by filtering human DNA and analysing bacterial genes. Finally, the bacterial species were cultured, the key genes were identified, and the statistical analysis was performed. Species richness and evenness metrics representing alpha diversity did not show significant differences between spaceflight and ground control groups. This means that spaceflight neither caused loss nor gain in overall microbial diversity. Additionally, the microbial community in both environments had a quite balanced species distribution as indicated by the Shannon and Simpson diversity indices. There were significant compositional differences as Principal Coordinate Analysis showed distinct clustering between spaceflight and ground control groups, thus reflecting notable changes in microbial composition. The clusters’ separation indicates that changes in the microbial community structure due to spaceflight environmental factors was one of the main reasons for the compositional differences. The *Firmicutes* and *Bacteroidetes phyla* dominated in both spaceflight and ground control groups which was evidently shown in the taxonomic composition section. This highlighted their key role in the gut’s homeostasis. On the other hand, spaceflight samples showed a significant rise in Proteobacteria level which may point to the microbiome experiencing stress or adapting to microgravity. A significant reduction of *Lactobacillus spp*. was found in space, exposed rodents which may have implications on host metabolism and immunity due to the decline of beneficial gut bacteria. Changes in microbial composition following the spaceflight have been evidenced; however, the microbiome diversity remained unaffected. Moreover, the variation in certain bacterial taxa could indicate the microbiome’s response to the space environment either by adaptation or stress.

Xihui Gan et al. (2024) studied the impact of mouse behaviour, gut bacteria, and gene activity under simulated microgravity conditions and determined their relationships with circadian rhythms. The mice were placed in noise and low-pressure conditions, normal conditions, and microgravity conditions. Hind-limb unloading was used to simulate microgravity conditions. C57BL/6 N mice were selected and divided into two groups: one housed under a simulated space capsule and the other in normal conditions. The simulated space capsule located at the Chinese Astronaut Research and Training Centre was employed in this study, which was temperature-, humidity-, and light-controlled. From day 7, noise and reduced atmospheric pressure were applied. The samples were collected by euthanising the animal and extracting the hypothalamus, liver and stool samples.

The expression of genes was confirmed by qRT, PCR technique using SYBR Green dye. DNA from stool was isolated, 16 S rRNA genes were amplified and sequenced, and the Microbiome composition was studied via QIIME, LEfSe, and KEGG pathway prediction. HIF1A expression was determined by immunohistochemistry using primary (GeneTex) and secondary (Bio, Rad) antibodies. The findings showed that after being subjected to low air pressure and noise, mice in the simulated space capsule exhibited an immediate reduction in locomotor activity that was still present in the subsequent phases. Mice kept in the animal room exhibited a slower rate of circadian re, entrainment compared to SSC mice, thus they may have experienced circadian disruption. In the hypothalamus, SSC and room animals exhibited alterations in global gene expression that were distinct from each other. The SSC mice had less differentially expressed genes as per their profile. KEGG analysis identified innate immune system, circadian rhythms, and prion disorders as main biological themes. Circadian genes showed significant changes in the animal room but remained largely unchanged in SSC. HIF1A levels suggested no hypoxic stress in SSC. Over time, gut microbiome adaptability decreased under SSC settings, while microbial diversity remained constant. The diurnal rhythms of specific bacterial genera were altered, and pathways associated with cardiovascular function were affected. In hindlimb-unloaded (HU) mice, a model of microgravity, the gut microbiota exhibited notable changes in the animal room but remained constant in SSC. While there was no significant difference in microbial diversity (α-diversity), the composition of the gut microbiota altered over time in AR, while these changes were reduced under SSC settings. Although some bacteria, such as *Adlercreutzia*, declined in SSC compared to AR, Bacteroidetes and Firmicutes remained the dominant phyla. *Prevotella* increased later in HU mice in SSC, demonstrating rhythmic bacterial changes induced by microgravity.

Ana Ramos-Nascimento et al. (Ramos-Nascimento et al. 2023) conducted a 6-degree angle Head-down tilt or bed rest study in collaboration with organisations such as NASA, DLR, and ESA to determine the effects of microgravity on the human gut microbiome. In this study, the participants were allowed to sleep in a slightly tilted downward position to simulate the microgravity (space) environment. The participants were divided into three groups: (A) Head rest group (HRG), (B) HRG + continuous countermeasures, such as exercise, given continuously throughout the study, and (C) intermittent countermeasures, sometimes given but not continuously. Stool samples were collected, and metaproteomics was performed. Data processing, including taxonomic and functional profiling, was performed. The results revealed that the countermeasures reduced microbial changes, and the microgravity simulation altered the composition of the gut microbiota, thereby increasing levels of *Clostridium* and Faecal bacterium. Drug-resistant and metabolic functional pathways were impacted, although indicators for bowel illness stayed constant. Centrifugation helped the formation of beneficial SCFAs by undoing changes in SCFA that were harmful. The Inverse Simpson Index. It is a metric for measuring diversity which is applied here for three different groups: A (green), B (red), and C (orange). The data presented show that the diversity of microbiota was quite similar between the different experimental groups, with both HDT & int CM exhibiting a marginally higher diversity level except at HDT50. It implies that intermittent countermeasures might be more effective in maintaining gut microbiota diversity than continuous countermeasures. The bed rest group demonstrated differential bacterial patterns and slightly altered gut bacteria compared to the normal group. Restaging in the wake of the changes was increased production of short, chain fatty acids such as acetate, butyrate, and propionate. Evaluating bacterial function, activity, and metabolite production will be a valuable measure of astronaut health and also the provision of countermeasures for the implementation of long space missions.

Ruqaiyyah Siddiqui et al. have investigated the changes in gut microbiota due to microgravity using the Hindlimb unloading C57/B16 mouse model for 20 days. At the conclusion of the experiment, the mice were sacrificed, and their gut tissues were taken for DNA and 16 S rRNA extraction to carry out metagenomic analysis. The findings demonstrate that the gut bacteria of the normal and microgravity, exposed mice were different. Normal mice exhibited a higher level of Bacteroidetes, whereas microgravity, exposed mice had an increased level of Firmicutes. Some beneficial gut bacteria like *Akkermansia muciniphila* were drastically reduced in microgravity. In total, 443 bacterial species were unique to microgravity, and 449 were different from those under normal gravity. Changes in gut flora could influence immunity, metabolism, digestion, and overall health. It is vital to understand such effects for the wellbeing of astronauts on long, term space missions (Siddiqui et al. 2022).

Yongtao Yang et al. investigated how microgravity affects gut bacteria and to what extent estrogen can protect against these changes. The findings indicated that microgravity altered gut bacteria, raised the detrimental bacteria, e.g. *E. coli*,* Bacteroides fragilis*, and *Fusobacterium nucleatum*, and lowered the beneficial bacteria *Bifidobacterium longum*. The bacterial changes thus resulting could weaken the gut barrier and facilitate the translocation of harmful bacteria from the gut to the blood. As part of the study, estrogen was given to mice and it was found that this helped in preventing bacterial overgrowth, particularly E. coli, and consequently reducing the markers of gut damage. Therefore, the study inferred that estrogen administration can be a measure for preventing gut inflammation and a means of reducing the occurrence of digestive problems of astronauts going on space journeys (Yang et al. 2017).

Yijuan Han et al. examined the gut microbiome of normal and simulated microgravity rats, where 16 S rRNA gene sequencing was also conducted. The microgravity conditions were simulated using the diamagnetic material magnetic levitation method. This study observed that under microgravity, the good bacteria in the gut decreased, while the harmful bacteria increased. Microgravity also changed bacterial metabolic pathways that are crucial for the bacteria, thus impacting pyrimidine metabolism, fatty acid metabolism, peptidoglycan biosynthesis, and carbon fixation. Detection of rare microorganisms can be greatly improved by using advanced equipment, such as PCR (Han et al. 2022). Zhujun Wu et al. (2024b) studied the gut microbes using a biorthogonal metabolic labelling method, where the bacteria were monitored non-invasively under the microgravity environment. The technique employs D-amino acid (3-azido-D-alanine, D-Ala-N3) and ICG-DBCO through bioorthogonal chemistry to selectively modify bacterial peptidoglycan without affecting mammalian cells. The effects of gut microbes were tested under normal conditions and during a 30° head-down tilt, simulating microgravity. The copper-free click chemistry was confirmed by mass spectrometry. In this research work, *E. coli* and *S. aureus* were labelled with Cy3-DBCO and analysed using confocal microscopy and flow cytometry. The rats were orally administered D-Ala-N_3,_ followed by administration of ICG-DBCO. Confocal imaging revealed distinct fluorescence on bacterial cell walls, confirming the metabolic incorporation of D-Ala-N3.

**Fig. 4:**
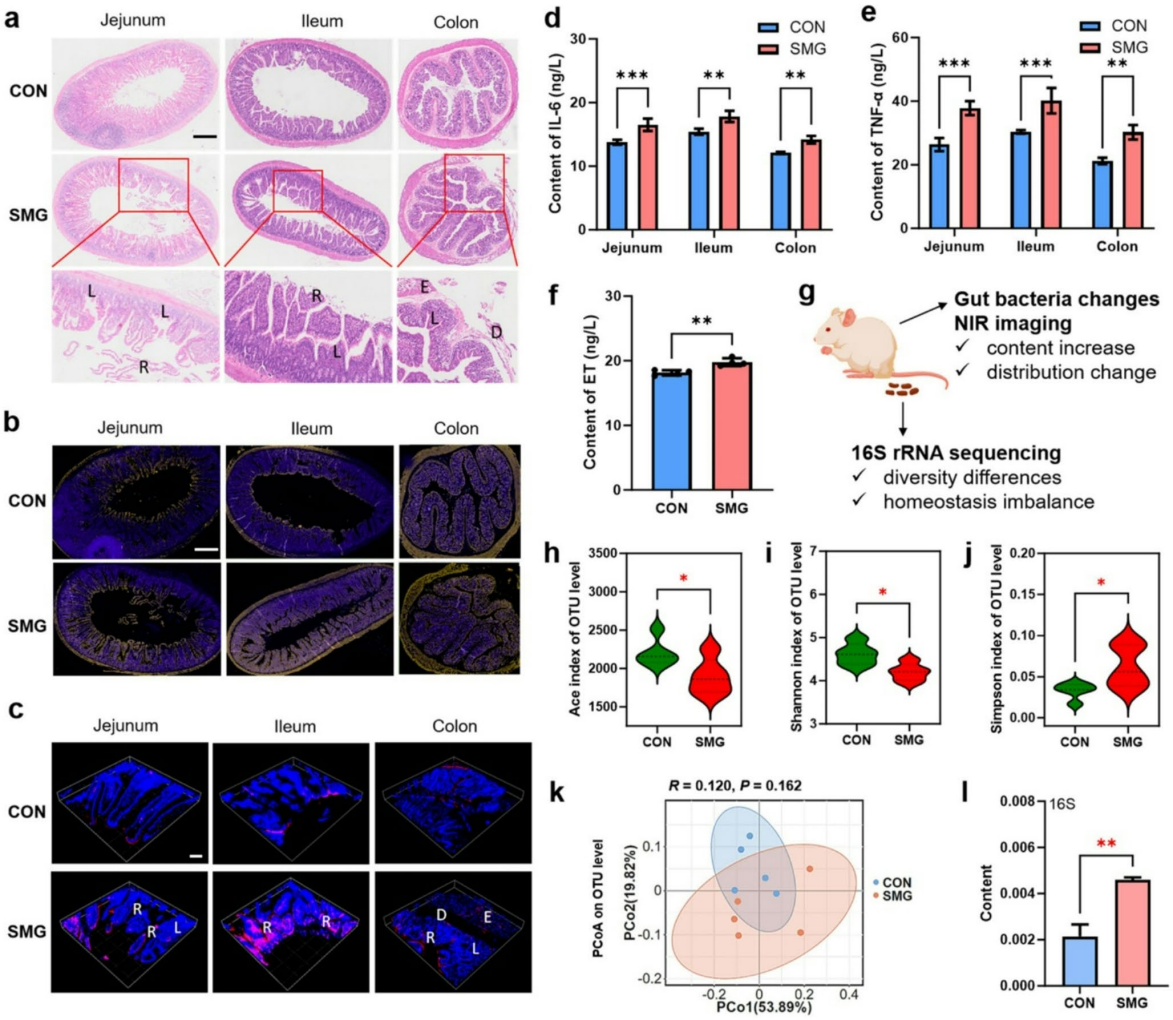
Effects of simulated microgravity (SMG) on intestinal pathology and gut microbiota. (a) H&E-stained sections show crypt loss, villus rupture, edema, and basal detachment in SMG vs. control. (b) Fluorescence images of gut bacteria (red) and nuclei (blue) across intestinal sections. (c) 3D confocal imaging of bacteria in intestinal tissues. (d–e) IL-6 and TNF-α levels in gut tissues (ELISA, *n* = 3). (f) Blood endotoxin levels indicate barrier permeability (*n* = 3). (g) Schematic of NIR imaging and 16 S rRNA sequencing. (h–j) α-diversity indices (Ace, Shannon, Simpson) show reduced diversity in SMG. (k) β-diversity differences via PCoA. (l) qPCR analysis of bacterial content; *p* = 0.0083 (Wu et al. 2024b)

Flow cytometry was used to confirm the high specificity and efficiency of the labelling method. After rat faecal samples were collected, gut bacterial strains *Bacillus subtilis*,* Lactobacillus murinus*, and *Bacillus licheniformis*, were evaluated. These bacteria showed very strong fluorescence signals when metabolically labelled, thus confirming the chemical modification was successful. Quantitative analysis manifested a large increase in fluorescence intensity, thus corroborating that this method is a viable technique for in vivo gut bacterial imaging. In microgravity, exposed rats, the fluorescence was six times higher, which indicates that the bacterial load was increased rather than the labelling efficiency. It was confirmed by in vitro studies that microgravity had no impact on bacterial labelling efficiency. Following metabolic labelling, rats were sacrificed, and their gastrointestinal tracts were subjected to analysis. Ex vivo imaging showed that the increase in fluorescence was most notable in intestinal regions, especially the ileum, in microgravity, exposed rats. With respect to normal conditions, the fluorescence in microgravity, exposed rats were more spread out, thus indicating a larger bacterial in the gut. Microgravity exposure caused severe tissue damage, such as the breakdown of microvilli, the loss of crypts, and the detachment of the colonic basal layer. The biorthogonal labelling of the intestinal tissue sections also showed an increase in gut bacteria in microgravity, exposed rats, especially in the ileum, which was in line with tissue damage. In order to study the impact of microgravity further, the levels of proinflammatory cytokines such as interleukin, 6 and tumor necrosis factor, alpha were also measured in the intestinal tissues. The level of these inflammatory markers in the microgravity, exposed rats was significantly higher than that of the control group, in addition to the finding of increased plasma endotoxin levels, which is a sign of a compromised intestinal barrier function. Figure 4 shows the total picture of the intestinal damage in the microgravity, exposed group, the resultant inflammatory response, and the disruptions in gut microbiota composition and diversity. The results of this study demonstrate that microgravity is highly likely to be associated with deterioration of gut health. The authors interpret their results as evidence that microgravity exposure impacts gut bacterial homeostasis negatively, which in turn leads to an increased bacterial load, intestinal inflammation, and barrier dysfunction.

**Fig. 5:**
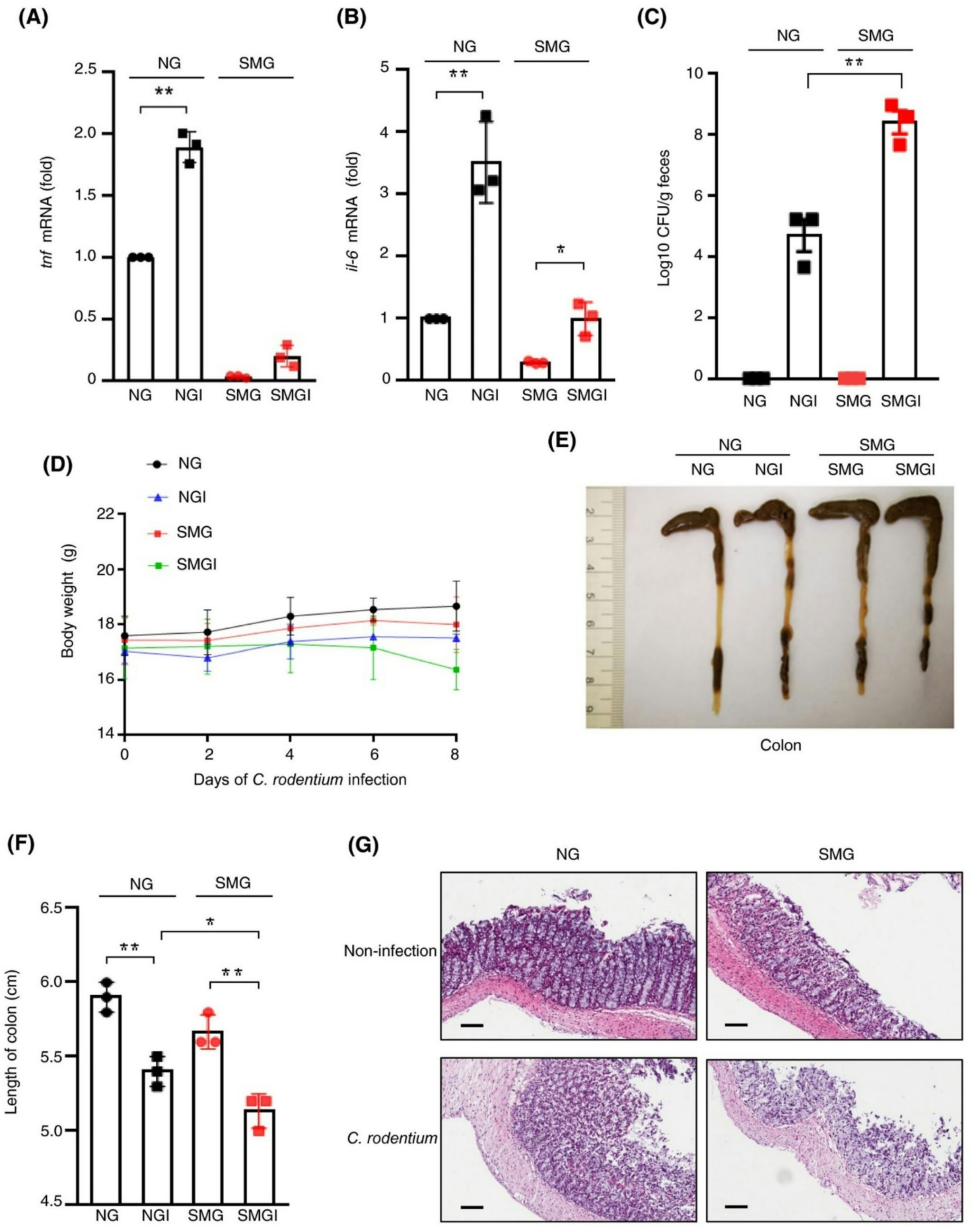
Simulated microgravity (SMG) enhances susceptibility to C. rodentium. (A–B) qPCR shows elevated Tnf and Il6 mRNA in colons of infected SMG mice. (C) Fecal CFUs of C. rodentium increase under SMG. (D) SMG-infected mice show greater weight loss. (E–F) SMG infection reduces colon length. (G) Histopathology reveals worsened colon damage under SMG. Reproduced with permission (Wang et al. 2020b)

Jing Wang and his colleagues conducted the research on immune response and gut microbiota in simulated microgravity conditions. They figured out that simulated microgravity severely depresses the secretion of inflammatory cytokines TNF, IL, 6, etc., in enteropathogenic E. coli infected macrophages, and the suppression was confirmed by qRT, PCR and ELISA. With the help of RNA sequencing, QRT, PCR, and western blotting, the researchers found out that SMG hampers the MAPK signalling pathway, especially the p38 and Erk1/2 pathways. Therefore, simulated microgravity may reduce the capability of macrophages to fight pathogens by suppressing the expression of key immune genes. Figure 5 shows that VSL#3 reverses the compromised immune response to pathogenic bacterial infection caused by simulated microgravity (SMG). qPCR measurement of Tnf and Il, 6 mRNA (A, B) indicates that SMG, infected (SMGI) mice show decreased cytokine expression, while VSL#3 administration (SMGVI) recovers immune response. Bacterial load (C) shows an increased number of C. rodentium CFUs in SMGI mice, but VSL#3 drastically lowers bacterial presence. Western blot analysis (D) shows a decrease in phosphorylation of Erk1/2, p38, and JNK signaling pathways in SMGI mice, and VSL#3 treatment recovers it. Colon length measurements (E, F) indicate shortening in SMGI mice as a result of inflammation, whereas VSL#3 keeps it at a normal level. Histopathological analysis (G) also supports that SMGI mice have very extensive colon damage that is relieved by VSL#3 treatment. In sum, these findings indicate that VSL#3 antagonizes SMG, induced immune suppression through the modulation of immune responses, bacterial clearance, and gut barrier protection (Wang et al. 2020b).

Yifan Wang et al. studied gut microbes through the hindlimb unloading microgravity model. The findings revealed that mice exposed to microgravity experienced poor weight gain, glucose intolerance, and insulin resistance. Beneficial gut bacteria, such as Bifidobacterium and Akkermansia muciniphila, have been depleted due to this exposure, and additionally, inflammation, related markers in the blood and the liver have been increased. Treatment with Bifidobacterium had counteracted the inflammation, enhanced glucose tolerance and facilitated the rebalance. Therefore, this research demonstrated a close association of gut microbiota with metabolism under space, like conditions, thus indicating the necessity to keep astronauts’ gut health under surveillance to avoid metabolic disorders. (Wang et al. 2019). Peng Jiang et al. examined the gut microbiome of mice on the International Space Station. During this 37, day experiment, mice were kept in a microgravity environment simulation. The scientists employed an innovative method, STARMAPs, to align their findings with those from other spaceflight investigations. The study indicates that space travel leads to an increase in microbial diversity and changes the composition of the gut microbiome, which is in line with previous space missions but differs from those caused by space radiation on Earth. Besides, several bacterial groups were altered, thus affecting their capacity for energy metabolism. This study also links such microbiome alterations to liver gene expression, particularly in protein metabolism. It is through these findings that scientists can trace the strong influence of space conditions on gut bacteria and metabolism. This work will lead to the development of solutions to maintain the health of astronauts during extended space missions (Jiang et al. 2019).

E. Gonzalez et al. examined how space travel affects the gut microbiome and body physiology of mice living on the ISS. They found that 44 strains of gut bacteria, among which were beneficial bacteria, have been decreased, whereas bile acid, and fat, producing bacteria were increased. Spaceflight led to an increase in total body weight of mice. The weight of the ISS mice had significantly gone up after 56 days. Contrary to muscle and bone loss typically seen in microgravity, the weight gain was found to be due to increased fat tissue and lipid accumulation. The study of gut microbiota revealed that the species composition changed due to spaceflight, for example, the level of *Parabacteroides goldsteinii* went up. The metabolism of short, chain fatty acids, the conversion of bile acids, and the potential of pathogenicity were among those affected by the changes. *D. welbionis*, which is a butyrate, producing bacterium associated with metabolic health, was more abundant, suggesting that spaceflight has the potential to alter gut microbiota and metabolic activities. Spaceflight induces major changes in the intestines such as ECM remodelling, changes in mucin, and immune suppression. The levels of collagens, laminins, and integrins are increased, while the levels of the main immune molecules and cytokines are decreased, which results in a weakened gut defence. Spaceflight on the liver perturbs several functions such as bile acid metabolism, cholesterol regulation, and detoxification. There are indications of mitochondrial dysfunction, cholesterol build up, and bile acid synthesis disorders, which reveal a state of metabolic stress and a breakdown in the gut-liver axis (Gonzalez et al. 2024).

### Radiation exposure

David Casero et al. conducted a study on space radiation effects on gut microbes by using male C57BL/6J mice. Animals were divided into two groups: one group of mice was brought to NASA’s Space Radiation Laboratory, and the other was kept under control conditions. After ionising radiation, mice were maintained under standard conditions with a normal diet and administered fenbendazole. Fecal samples were collected post, irradiation which were then divided for sequencing and metabolomics work. Data indicated that lower doses (0.1, 0.25 Gy) of ionising radiation caused more significant changes to gut microbial composition and function than higher doses (1 Gy). This sort of threshold response is consistent with the phenomena of hyper, radiosensitivity and induced radio-resistance observed in mammalian cells. Low doses elicit the protective responses, while at higher doses, DNA repair mechanisms get activated. The findings indicated that radiation long, term altered the overall gut microbial composition, therefore, favouring harmful species such as *Erysipelotrichaceae* and *Akkermansia muciniphila* while decreasing beneficial bacteria such as Firmicutes and Actinobacteria. Phosphonate metabolism and lipopolysaccharide production were among the metabolic pathways that showed functional alterations. Metabolomics analysis resulted in declines in essential metabolites such as phosphatidic acid, leukotriene B4, and tryptophan derivatives, thus pointing to a strong association among oxidative stress (Casero et al. 2017).

Noopur Gupta et al.(2020) studied the effect of radiomitigators on gut microbes. A radiation dose of approximately 1 Gy/min was administered to C57BL/6J mice. The animals were grouped as follows: Group A received irradiation; Group B received whole-body radiation at 7 Gy/min; Group C received radiation; Trichostatin A (radiomitigators) was administered i.v.; and Group D received only the drug without radiation. The results revealed that the irradiation group of animals experienced a reduction in body weight, followed by an increase. The untreated group has increased body weight. The drug-treated group has experienced increased body weight. Irradiated animals that received Trichostatin A and radiation experienced an initial weight loss on day 1 but recovered more rapidly than the irradiation group. By day 14, these animals had gained body weight, demonstrating the potential mitigating effects of Trichostatin A against radiation-induced weight loss. The radiation group lowered the numbers of bacteria in the small intestine and ileum, whereas the drug, treated group experienced accelerated bacterial recovery by day 7. The radiation group had a large number of bacteria spreading to different organs, e.g., the liver, spleen, and mesenteric lymph nodes by day 7. Trichostatin A lessened the translocation, mostly in the liver and Mesenteric Lymph Nodes. On day 3, In the radiation group, aerobic bacteria such as *Pseudomonas mendocina* were present, which was postponed until day 5 (drug, treated group). *Lysinibacillus sphaericus* as an anaerobic bacterium has been found by day 7 in the group exposed to radiation, whereas it was still very low in the drug, treated group. Radiation has caused the acceleration of gut transit, and the drug has avoided this effect, thus keeping the normal small intestinal transit. Histopathology showed villi shrinkage in the radiation group by day 5, which is a sign that the drug has preserved villi integrity. Therefore, the findings indicate that Trichostatin A alleviated radiation, induced gut damage, reinstated the microbiota, and lessened bacterial translocation.

Xiaozhou Zeng et al.(2022) studied the effects of radiation on gut microbes in the C57BL/6J mice model. The mice were exposed to 13 Gy of radiation, housed for 12 h, and provided with proper food. Faeces were collected from mice every 2 days. The mice were administered antibiotics for two weeks. Faecal microbiota transplantation was done by administering faeces to the mice. Bacteria such as *L. murinus and A. muciniphila were administered to the mice by* oral gavage. Bacteria labelled with the DIR dye were also administered by oral gavage. The DNA was extracted from the faecal sample, and qPCR was performed. The protein analysis was also performed using the ELISA technique, and Histology was conducted on intestinal tissue. As a result of this research work, the dominant mice experienced more severe intestinal radiation injury than the subordinate mice, characterised by lower survival rates and higher inflammation.

**Fig. 6:**
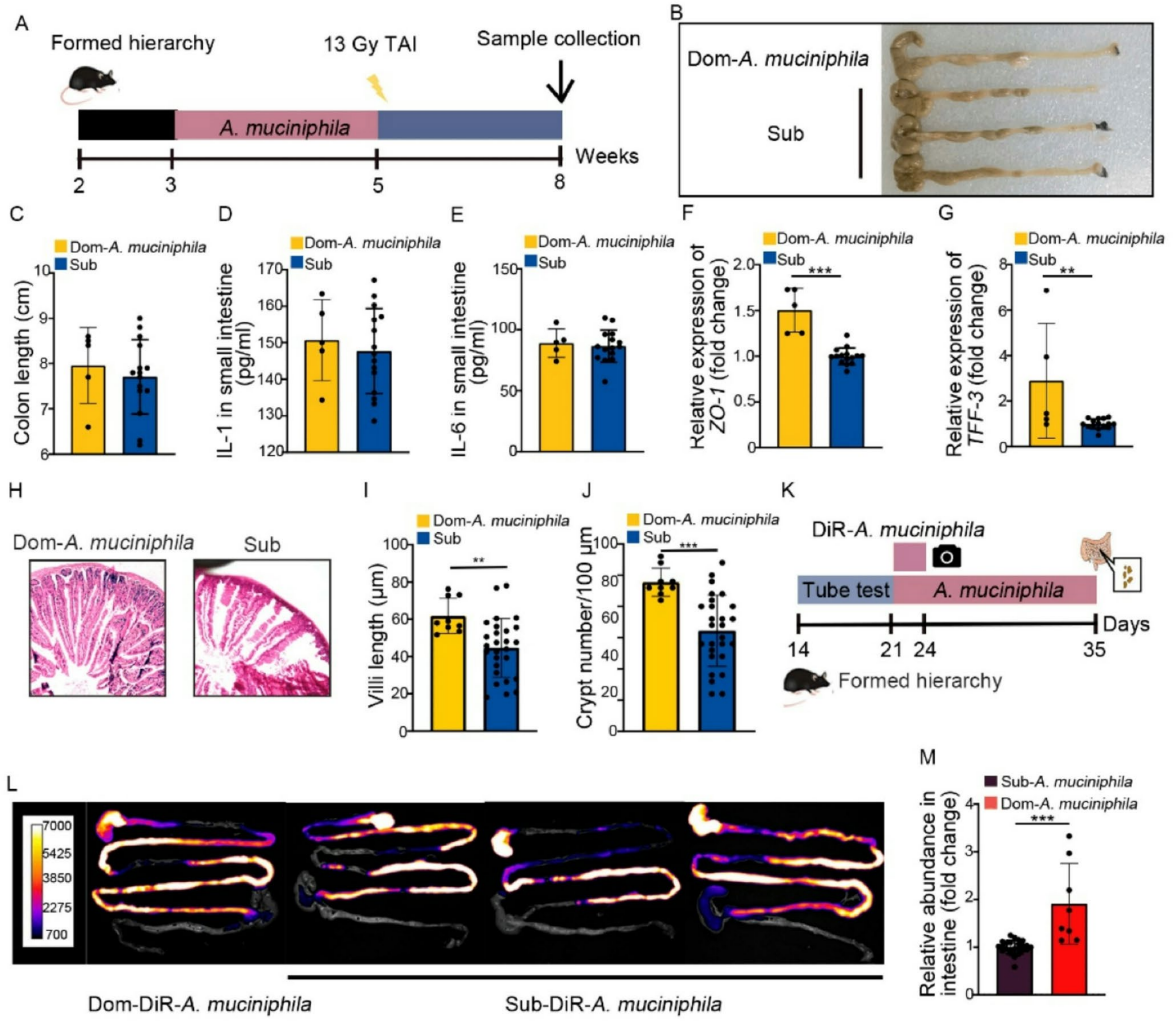
A. muciniphila protects dominant (Dom) mice from intestinal radiation injury. (A, K) Experimental setup. (B–C) Treated Dom mice show preserved colon length. (D–E) IL-1 and IL-6 levels are reduced. (F–G) Barrier proteins ZO-1 and TFF-3 are increased. (H–J) Histology confirms improved villi length and crypt numbers. (L) Fluorescence imaging verifies stable colonization of A. muciniphila (Zeng et al. 2022)

Dominant mice exhibited significant alterations in gut microbiota following irradiation, characterised by reduced α-diversity and distinct shifts in bacterial composition compared to subordinate mice. The antibiotic treatment eliminated microbial differences and equalised radiation injury between dominant and subordinate mice. Faecal microbiota transplantation from subordinate mice to dominant mice reduced radiation toxicity in dominant mice, indicating the microbiota’s key role. Lactobacillus murinus reduced radiation toxicity in non-hierarchical mice but failed to protect dominant mice due to poor gut colonisation, as shown in Fig. 6. *Akkermansia muciniphila* stably colonised in dominant mice and significantly mitigated their radiation injury, making it a more effective probiotic for high-status individuals. muciniphila in Dom mice, while q-PCR analysis in (M) confirms its higher relative abundance compared to subordinate mice. The overall findings suggest that *A. muciniphila* plays a crucial role in mitigating radiation-induced intestinal damage in dominant mice. Finally, this study concluded that a personalised probiotic treatment strategy will be effective in treating radiation-induced intestinal damage.

**Fig. 7:**
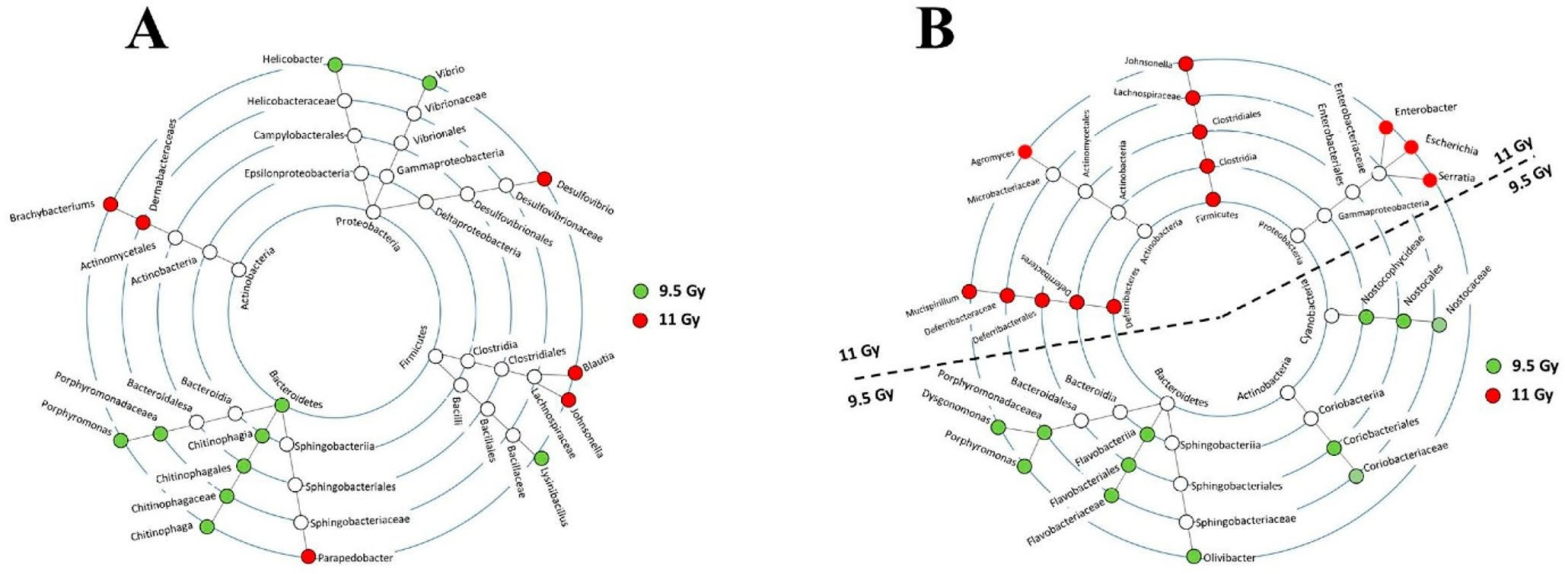
Taxonomic cladogram showing bacterial abundance profiles. Concentric rings represent taxonomic ranks from phylum to genus. Nodes are color-coded: green (9.5 Gy), red (11 Gy), white (unchanged). (A) Day 3 post-TBI; (B) Day 9 post-TBI (Zhou et al. 2024a)

Gui-qiang Zhou et al. (2024a) investigated the effects of radiofrequency radiation on the gut microbiota and brain. The results revealed that radiofrequency radiation (RFR) (9.5 Gy and 11 Gy) exposure induced anxiety-like behaviour, as shown by decreased time spent in the centre of the open field test and reduced open arm entries in the elevated plus maze. Radiofrequency radiation exposure led to neuronal pyroptosis in the CA3 region of the hippocampus, rather than autophagy or apoptosis, as indicated by increased GSDMD protein expression. The pyroptosis was NLRP3-dependent, with elevated levels of Cleaved caspase-1 and GSDMD-N. Radiofrequency radiation exposure changed gut microbiota composition and consequently caused metabolic disorders in faeces, serum, and brain tissue, which affected the metabolic pathways of lipids, neurotransmitters, and inflammation. Correlation analysis showed that changes in gut microbiota were connected to metabolic alterations, which indicated that radiofrequency radiation effects involved a gut-brain interaction. Nabarun Chakraborty et al. (2024) studied the effects of radiation on gut microbes in mice. The mice in the study were irradiated with a high dose and euthanized after that. The gut contents were collected and analysed. The findings indicated alteration of gut microbial composition. More beneficial bacteria, like *Firmicutes*, were found after the treatment, while harmful inflammation causing bacteria, such as *Deferribacteres* were increased. Figure 7 depicts the impact of lethal radiation doses (9.5 Gy and 11 Gy) on gut microbiota in CD2F1 male mice, showing shifts in bacterial composition (*Firmicutes* increase, *Bacteroidetes* decrease), microbial diversity changes (PCoA, Simpson’s and Chao1 indices), and specific taxa enrichment (LEfSe analysis), with time post-irradiation. Further analysis revealed that the bacteria were activating genes involved in fat metabolism and energy production, but these processes were suppressed in the rest of the body. This suggests that the normal balance between gut bacteria and the host’s metabolism was disrupted after radiation. This study concluded that radiation could cause a severe imbalance in gut bacteria. Further studies are required to prevent these imbalances during space travel.

## Countermeasures for maintaining gut health in space

### Exercise

Various studies have demonstrated that exercise or physical activities may alter gut health. Exercise increases gastrointestinal motility and blood flow, thereby altering the intestinal environment, including pH, bile production, and oxygen tension. Thus, this may lead to alterations in gut microbial composition (Mailing et al. 2019; Valder and Brinkmann 2022; Varghese et al. 2024). It has been observed that beneficial bacteria increased and pathogenic bacteria decreased. Beneficial bacteria will increase SCFA levels, leading to improved tight junction protein expression, decreased leaky gut, and reduced translocation of endotoxins such as lipopolysaccharides. Enhanced local and systemic inflammation and reduced anti-inflammatory cytokines (Welcome 2019; Daily et al. 2021; Wang et al. 2021b; Cullen et al. 2023). Even though there are various gut health benefits associated with exercise, such as improved metabolic health, enhanced brain function, and greater protection against infectious diseases. The role of gut health in terrestrial human and animal studies has been explored, but in microgravity or spaceflight conditions, these studies are limited. These studies may provide countermeasures for astronauts and space travellers to safeguard their gut health during and after space travel. The following table presents case studies on the effects of exercise on gut health in terrestrial conditions, which can also be utilised during space travel to maintain gut health. From these case studies, it can be hypothesised that aerobic exercise may help protect and support gut health during space travel under microgravity conditions. But high-intensity exercises may require larger spaces to perform, whereas moderate-intensity exercises can be performed to overcome these limitations. Case Studies on the impact of exercise on gut microbiota is given in Table 1.

**Table 1:** Case Studies on the impact of exercise on gut microbiota

Si. No.	Study design & population	Type of exercise	Duration of exercise	Key Findings	Reference
1	Humans; age: 12–14 years; population: 224 adolescents	Aerobics	Participants ran at 50%–70% of maximum heart rate for 30 min/day, 4 days/week, for 3 months.	The results revealed no significant effect on gut microbiota in clinically healthy or subthreshold adolescents, though beta diversity may be impaired in the latter.	(Wang et al. 2021a)
2	Female football players; age: 11–14 years; population: 29	Football drills, tactical exercises, interval running, strength training, and dynamic stretching.	Moderate exercise: trained 5 times/week for 30–40 min at 40–59% heart rate reserve (≥ 150 min/week); vigorous exercise: trained 3–4 times/week for 30–35 min at ≥ 60% heart rate reserve (≥ 90 min/week).	This study revealed that moderate exercise boosted gut diversity, while high-intensity exercise caused microbial shifts, suggesting the need for personalised training and nutrition strategies.	(Yang et al. 2025b)
3	Humans; age: 18–25 years; population: 18	Gym exercise, walking, ball games, running	Days: 3–15; 1–6 times per week; duration: 1–2 h or less; MET: 2–12.	This study revealed that exercise improved bone health by altering gut microbiota and boosting butyric acid, which supports bone strength.	(Dou et al. 2025)
4	BL6 mice; age: 20 months old	Treadmill exercise	30 min/day, 5 days/week for 8 weeks.	This study revealed that Treadmill exercise increased Lactobacillus and Bifidobacterium levels in old mice; it had no significant effect on restoring A. muciniphila levels, and it partially reversed age-related gut microbiota changes.	(Park et al. 2024)
5	C57BL/6J mice; age: 4 weeks	Treadmill	30 min/day at 12–15 m per minute for 4 weeks	This study showed that exercise enriched beneficial gut microbes (Akkermansia, Butyricimonas) and improved heart-related microbiota profiles, while Helicobacter was associated with worse cardiac function.	(ALLEN et al. 2018)
6	Female SD rats; weight: 180 g; population: 40	Motorized wheel	1 h twice daily (at 9 am and 3 pm)	The composition and function of the gut microbiota were altered by regular exercise, thereby decreasing depressive-like behaviour.	(Liu et al. 2017)
7	Male C57Bl/6 mice; population: 40	Treadmill	The training lasted 30 min per day, five days a week, over eight weeks.	These results revealed that 8 weeks of low-to-moderate exercise is not sufficient to significantly alter the gut microbiota in the presence of a high-fat diet.	(Sheng et al. 2021)
8	Healthy elderly; age: 62–76 years; population: 33	Aerobic cycling	30 min for the first 2 weeks and 45 min for the final 3 weeks	The diversity and composition of the gut microbiota are not significantly altered by short-term endurance exercise, but certain functional changes have been observed.	(Taniguchi et al. 2018)
9	Male C57BL/6 N mice; age: 4-weeks-old mice population: 60	Treadmill	2-minute runs at 15 m/min; repeated 10 times daily for 8 weeks	The results revealed that exercise preconditioning may improve survival by balancing immune responses and modifying gut microbiota.	(Kim and Kang 2019)

### Probiotic

One of the probiotics helping immunity, pathogen antagonism, and GI health, Lactobacillus acidophilus ATCC 4356, was tested by Castro and Wallace (2017) in a rotating-wall vessel bioreactor that simulated low-shear, modelled microgravity (LSMMG) under anaerobic conditions of the human gut. This strain showed no significant differences vs. controls in growth curves (log phase at 2 h, stationary at 16 h, pH ~ 4.4), survival through simulated gastric (pH 2 pepsin) or intestinal (pH 8 pancreatin/oxgall) juices over 120 min, or transcriptome profiles (RNA, Seq of 35 M reads across 1807 genes; *r* = 0.95 correlation, no FDR < 0.05 differentials) even though, unlike previous aerobic or pathogen studies that showed altered growth/stress response, it was the first time that the strain was tested anaerobically. From the results of the work, L. acidophilus is expected to behave like Earth even in spaceflight, thus it can be considered a non-invasive countermeasure for astronauts’ microbiome/immune dysregulation due to sterile space diets and mission stresses; anaerobic LSMMG may prevent pathogens, like enhancements, probably because oxygen is involved (Castro-Wallace et al. 2017).

Yim et al. (2020) studied EcN, a probiotic bacterium known for promoting gut health and repair, and a significant constituent of products like Mutaflor designed for cases of gut disorders such as constipation and Crohn’s disease. They did this by emulating the effects of space microgravity (MG) using a spinning clinostat device and normal gravity. The experiment showed that EcN proliferated more slowly in MG, and the researchers went on to analyse its genes using RNA-seq at two stages: early (logarithmic) and late (stationary) growth. Major problems included: Obtaining of biofilm components initially enhanced as a defense mechanism facilitator, but later there was reduced attachment of gut cells (adhesion genes such as focA and the ecp operon were repressed) being susceptible to stomach acid (downregulation of the hde and gad acid, resistance genes) less uptake of essential metals like iron, zinc, and copper, andBeing affected in sugar/energy metabolism as a result of “starvation zones” which are nutrient, poor. While pathogenic organisms benefit from changes occurring at MG, EcN limitations indicate its ability to survive would be diminished in the guts of astronauts. Hence, there is a need for space-adapted probiotics to maintain microbiome health during long-term space missions, as identified by this research. (Yim et al. 2020).

Dongyan Shao et al. studied the effects of microgravity exposure on *Lactobacillus acidophilus* probiotics. The results revealed that the simulated environment didn’t alter the shape of the bacteria; however, their growth was faster, and they became more tolerant of acidic conditions (even at very low pH) and bile at low levels. It became less sensitive to certain antibiotics. Its ability to adhere to gut-like cells (Caco-2 cells) remained unchanged. The bacteria and their liquid phase showed more potent antibacterial activity against harmful bacteria such as *Salmonella* and *Staphylococcus*. It also improved its ability to lower cholesterol in lab tests by influencing genes involved in cholesterol metabolism in liver cells (Shao et al. 2017).

Thus, these studies revealed that more research on probiotics is required to utilise these species in the space environment. Spaceflight’s microgravity, radiation, isolation, and diet disrupt female astronauts’ microbiomes, reducing beneficial Bifidobacterium/Lactobacillus while increasing pathogens such as E. coli, thereby raising the risks of infection, bone loss, cancer, and immune dysfunction, which also differ by sex. The use of probiotics (e.g., L. rhamnosus GR, 1, L. reuteri RC, 14) is an option to antagonize this effect by preventing UTIs (with the same impact as antibiotics without resistance), helping vaginal and reproductive health, limiting breast cancer, increasing bone density, reducing stress/IBS, and facilitating viral clearancean area apart from which female, specific trials are needed for Mars expeditions (Urbaniak and Reid 2016).

## Clinical trials

Yakult Honsha and JAXA were the sponsors of the randomised, open-label trial NCT02618005, which enrolled 10 healthy astronauts (aged 25–80) who had been on the ISS for 3 + months. The purpose was to determine whether administering *Lacticaseibacillus paracasei Shirota* (LcS) probiotics during spaceflight would alleviate disturbances in immunity and the gut microbiota caused by the space environment. The LcS group was given five freeze-dried capsules daily for four weeks prior departure (R, 4 W to R, 1d), and the control group was not given any. Over 9, 18 months (pre, in, post, flight) time window, faecal LcS was quantified by PMA and qPCR; immune markers in blood/saliva were determined by ELISA/bioassay; and microbiota composition was analyzed by RT-qPCR, qPCR plus next-gen sequencing. The trial began in October 2015 and concluded in April 2022; however, the results have not yet been disclosed. Users of antibiotics/laxatives, or those with conflicting studies, were excluded from the study.

INFANT Intensive Protocol NCT Flights: Liftoff 2 Study in Long duration (2 Weeks for 55–65 years old) (1) Risks: Microgravity, including effects on muscles, bones, balance, heart concentric changes, and cognitive (brain) deterioration during 2 weeks of head-down bed rest (microgravity simulation) in young and old (55, 65 years), 24 subjects, 12 men/12 women. (2) Intervention: Exercising to counteract these effects. Participants stayed 26 days at the centre: 5 days adapting, 14 days in 6 tilted bed rest (head down), and 7 days recovering. During bed rest, the training group performed aerobic/strength exercises; controls remained inactive (randomised, open label). Measured changes in cognition (NIH tests, questionnaires), mood (scales), brain (MRI), muscle/fat (body MRI), heart (MRI), bones (CT scans), strength (dynamometer/jumps), balance/orthostatic tolerance (tests), blood vessels (ultrasound), protein turnover, sleep (EEG), frailty, gut/oral microbes (sequencing), performance (walk tests), bone/cytokine markers (ELISA/multiplex), and more baseline, bed rest, recovery. Completed; pubs show exercise cuts insulin resistance, protects executive function, and links neural injury to deconditioning. Funded by CSA/CIHR; healthy, smokers only, no major illnesses.

France’s space agency CNES conducted trial NCT03915457 on 20 healthy males (aged 20, 45) in order to see if thigh cuffs can prevent deconditioning as a result of 5 days of dry immersion water bath that mimics weightlessness’ fluid shifts, loss of support, weakening of muscles/bones, heart problems, and eye issues such as Spaceflight, Associated Neuro, ocular Syndrome (SANS) due to headward fluid buildup. Randomised open-label: one group did plain dry immersion (up to the neck in thermoneutral water under a fabric); the other wore thigh cuffs (elastic strips at ~ 30 mmHg pressure) for 10 h/day (8 am, 6 pm) during immersion to trap fluid in the legs, like Russian cosmonaut bracelets. Primary: optic nerve sheath diameter (echography) as an intracranial pressure marker. Secondaries: eye fiber thickness (OCT), brain structures/veins (MRI/gadolinium), perfusion (scintigraphy), eye pressure (tonometry), orthostatic tolerance (LBNP), body fluids (bioimpedance), blood pressure rhythms, cephalic fluid shifts (ultrasound), metabolism (RQ/post, meal), muscle biopsies (proteins/transcriptome/contractile properties/fat invasion), bone markers (ELISA), disc water/GAG (MRI spectroscopy), plus gut microbes (16 S sequencing), iron/hepcidin, cognition (VR tasks), body image. Baseline, during/after 5 days; fit, active, non-smokers, no diseases.

IU University of Applied Sciences’ ClinicalTrials.gov trial NCT06133530 intends to explore if daily 5.5 g human milk oligosaccharides (HMOs)undigestible prebiotics from breast milk that nurture beneficial gut bacteria such as bifidobacteria, increase production of short, chain fatty acids, lower inflammation, and support immunity can mitigate the stresses of Antarctica winter over at Concordia Station (3200 m, ~ 12 months isolation/hypoxia simulating spaceflight’s immune weakening, glucose issues, bone loss) in a triple, blind randomized parallel study, healthy 18, 65yo adults are given either HMO powder or maltose placebo orally; measurement times include baseline, every 1, 2 months during stay (months 4, 10), and 6, 7 months post, return, and encompass fasting blood, OGTT for glucose/insulin tolerance, saliva for stress hormones/viruses, feces for microbiota (16 S NGS), calprotectin/SCFAs, cytokines/CRP/lipids/bone markers/vitamin D/tryptophan metabolites such as serotonin/GABA, also mood/physical activity questionnaires. Started 2023, ongoing, safe per EU standards. Table 2 consists of the collection of clinical trial results of probiotics, exercise, thigh cuffs, and prebiotics that have been used as countermeasures to microgravity/analog deconditioning impacts such as immune/gut changes in astronauts or Earth simulations.

**Table 2:** Clinical trials on spaceflight-induced gut microbiota changes

NCT number	Study title	Sponsor	Locations	Start date	End date	Interventions	Brief Summary	Outcome	Reference
NCT02618005	Probiotics on Immune Function and Intestinal Microbiota in Astronauts Under Closed Microgravity Environment	Yakult Honsha Co., LTD	Japan Aerospace Exploration Agency, Ibaraki, Japan	2015-10	2023-03	**Dietary Supplement: Probiotic capsule**	Shirota (LcS) has shown benefits in supporting both. This study explores how spaceflight and consuming LcS on the ISS influence astronauts’ immunity and gut health.	LcS intake maintained faecal LcS levels, supported immune markers, and stabilised the gut microbiota during spaceflight.	(Yakult Honsha 2021)
NCT04964999	Understanding the Negative Effects of Bed Rest and Using Exercise as a Countermeasure	McGill University Health Centre/Research Institute of the McGill University Health Centre	Royal Victoria Hospital - Glen site, MontrÃ©al, Quebec, H4A 3J1, Canada	12-07-2021	2022-04	**Exercise**	24 adults (55–65 yrs) in a 26-day study at MUHC: 5-day adaptation, 14-day 6° bed rest, 7-day recovery; 12 with exercise, 12 controls. Four visits: screening, bed rest, two follow-ups. Assessments include sensorimotor, muscle, bone, cardiovascular, cognition, and biological samples.	Head-down bed rest (HDBR) induced widespread adverse effects on cognition, emotional health, brain and muscle structure, cardiovascular function, bone health, physical performance, sleep, and microbiome composition. At the same time, exercise countermeasures significantly mitigated many of these impacts.	(2021)
NCT03915457	Thigh Cuffs to Prevent Deconditioning Induced by 5 Days of Dry Immersion (DI-Cuff)	Centre National d’Etudes Spatiales	Medes-Imps, Toulouse, 31,400, France	18-11-2018	23-03-2019	**Other: Dry immersion Control Group. Other: Thigh cuffs intervention**	The study aims to evaluate whether thigh cuffs can mitigate deconditioning and fluid shift-related ophthalmological issues during 5 days of dry immersion in 20 healthy males, assessing multiple body systems and countermeasure effects.	Multiple physiological and biochemical changes in the eye, brain, muscle, bone, fluid balance, and cardiovascular systems were measured before and after five days of dry immersion.	(2019)
NCT06133530	Human Milk Oligosaccharides (HMOs) and Gut Microbiota, Immune System in Antarctica	IU University of Applied Sciences	IU International University of Applied Sciences, Erfurt, 99,084, Germany	24-09-2023	2027-08	**Dietary Supplement: Human milk oligosaccharides. Dietary Supplement: Maltose**	HMO blend given to verum group, placebo to control; samples (blood, saliva, faeces) and glucose test taken pre-, every 2 months during, and post-Antarctica stay; aims: modulate microbiota, reduce inflammation, improve immunity, glucose tolerance, nutrition, and neurotransmitters.	The study tracks changes in faecal biomarkers, gut microbiota, saliva hormones, blood inflammation markers, CRP, lipid metabolism, glycated albumin, GLP-1, and Fetuin-A over a 12-month Antarctic stay and post-return period.	(2023)

## Future prospects

A long-term mission for an astronaut profoundly alters the microbiome in the gastrointestinal tract. This alteration causes dysbiosis with an imbalance between the harmful and beneficial microbiome, which affects the immune system, causes physiological disruption, inflammation, and metabolic disorders. These health hazards and limited medical interventions are especially concerning for astronauts travelling on longer-term missions, such as to the Moon, Mars, and beyond. So, this understanding of the astronaut gut microbiota’s behaviour in Low Earth Orbit (LEO) has been explained in the above context. By understanding the mechanism of this alteration, scientists and industries are working to develop targeted solutions. Some specialised interventions are microbial approaches, dietary optimization, and advanced technologies (non-invasive and longitudinal monitoring studies) (Voorhies and Lorenzi 2016; Tesei et al. 2022). The microbial interventions are the targeted probiotic strains such as *Akkermansia muciniphila*,* Butyrate producers*,* Lactobacillus*, and *Bifidobacterium*. This probiotic helps to prevent and strengthen the gut microbiota, reduces oxidative stress, and improves gut barrier function. Fibre-rich prebiotics support the growth of SCFA-producing bacteria, and studies by the International Probiotic Association indicate that combining probiotics with prebiotics enhances metabolic health and microbial immunity (Voorhies et al. 2019; Bharindwal et al. 2023).

Fibre is one of the crucial dietary components that support SCGA production to maintain microbiota health and gut barrier integrity. So, the space mission food supply should be rich in fibre derived from plants to mitigate reductions in microbial diversity. Recent research focuses on developing an AI-based personalised nutrition program (Space ABC) to design individualised diets for astronauts based on microbiota profiles and metabolic requirements (2022).

Many ongoing research projects on the International Space Station (ISS) involve longitudinal studies that collect biological samples from astronauts at regular intervals to observe dynamic changes in the gut microbiome and related biomarkers. Astronauts on longer-duration missions have shown changes in the gut microbial community that persist throughout the mission (Zhou et al. 2024b). With this previous technological process, recent advancements in developing non-invasive monitoring by automated devices and wearable sensors have been made. This device will continuously monitor changes in biomarkers generated during metabolic and microbial disorders. Furthermore, this will not only investigate the role of microgravity on gut microbiota but also lead to an analysis of personalised interventions, such as probiotic supplements and dietary supplements (Awad et al. 2021).

## Conclusion

The gut microbiome plays a vital role in maintaining the astronaut’s health throughout the mission. As they face unprecedented challenges, such as microgravity, galactic and cosmic radiation, and prolonged confinement, these will synergistically cause dysbiosis. The depletion of beneficial microbiome causes these disruptions, reduced levels of neuroprotective metabolites, and elevated proinflammatory cytokines. Further, the gut-brain axis can impact the astronaut’s psychological well-being and neurological function. This will put the astronaut’s health at risk, leading to immune system dysfunction, loss of bone mineral density, and metabolic disorders. So, the essential requirements for understanding this complex mechanism in space conditions are space analogues and ground-based studies. These simulated conditions will reveal consistent patterns of gut microbiota, dysbiosis, and psychological stressors under space conditions, underscoring the importance of habitat and diet design in sustaining gut microbiome stability. The targeted dietary strategy for the astronaut to address this gut microbiota dysbiosis is to consume a high-fibre diet, probiotics, and prebiotics, which support the growth of beneficial gut microbes. However, for the preparation of missions beyond Low Earth Orbit (LEO) and extended missions, such as the Moon and Mars missions, continued research using ground-based simulated platforms is necessary to develop efficient strategies and countermeasures that ensure the well-being and productivity of astronauts in space.

## Abbreviations

GPR43 & GPR41: G-protein-coupled receptors
HDT: Head-down tilt
ISS: International Space Station
LET: Linear energy transfer
LPS: Lipopolysaccharides
LSMMG: Low shear modelled microgravity
NASA: National Aeronautics and Space Administration
RWV: Rotating Wall Vessel
SCFA: Short-chain fatty acids
SME: Simulated microgravity environment
TLR4: Toll-like receptor 4
LEO: Low Earth Orbit
ISS: International Space Station
SCFA: Short-chain fatty Acids
HDBR: Head-down bed rest
RFR: radiofrequency radiation
